# The Mechanism of Formation of Active Fe-TAMLs Using HClO Enlightens Design for Maximizing Catalytic Activity at Environmentally Optimal, Circumneutral pH

**DOI:** 10.1021/acs.inorgchem.3c00104

**Published:** 2023-03-27

**Authors:** Parameswar Pal, Marcus C. Schafer, Michael P. Hendrich, Alexander D. Ryabov, Terrence J. Collins

**Affiliations:** Department of Chemistry, Institute for Green Science, Carnegie Mellon University, 4400 Fifth Avenue, Pittsburgh, Pennsylvania 15213, United States

## Abstract

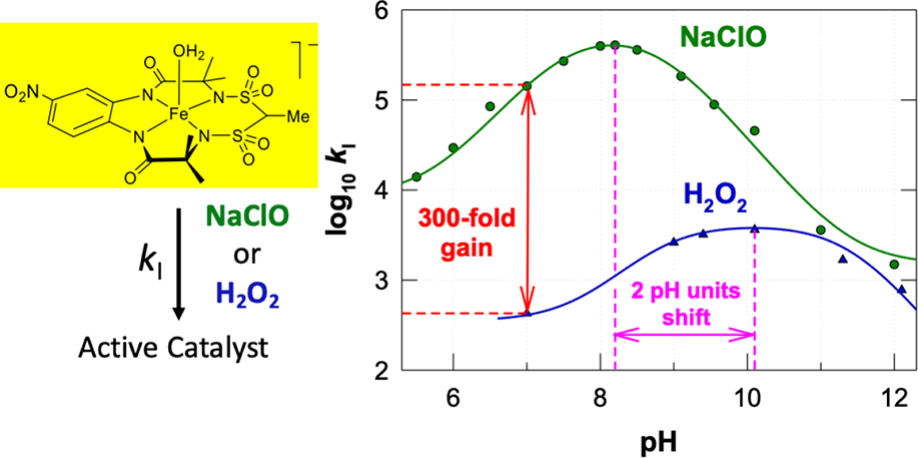

Fe-TAML/peroxide catalysis provides simple, powerful, ultradilute approaches for removing micropollutants from water. The typically rate-determining interactions of H_2_O_2_ with Fe-TAMLs (rate constant *k*_I_) are sharply pH-sensitive with rate maxima in the pH 9–10 window. Fe-TAML design or process design that shifts the maximum rates to the pH 6–8 window of most wastewaters would make micropollutant eliminations even more powerful. Here, we show how the different pH dependencies of the interactions of Fe-TAMLs with peroxide or hypochlorite to form active Fe-TAMLs (*k*_I_ step) illuminate why moving from H_2_O_2_ (p*K*_a_, ca. 11.6) to hypochlorite (p*K*_a_, 7.5) shifts the pH of the fastest catalysis to as low as 8.2. At pH 7, hypochlorite catalysis is 100–1000 times faster than H_2_O_2_ catalysis. The pH of maximum catalytic activity is also moderated by the p*K*_a_’s of the Fe-TAML axial water ligands, 8.8, 9.3, and 10.3, respectively, for [Fe{4-NO_2_C_6_H_3_-1,2-(NCOCMe_2_NSO_2_)_2_CHMe}(H_2_O)_*n*_]^−^ (**2**) [*n* = 1–2], [Fe{4-NO_2_C_6_H_3_-1,2-(NCOCMe_2_NCO)_2_CF_2_}(H_2_O)_*n*_]^−^ (**1b**), and [Fe{C_6_H_4_-1,2-(NCOCMe_2_NCO)_2_CMe_2_}(H_2_O)_*n*_]^−^ (**1a**). The new bis(sulfonamido)-bis(carbonamido)-ligated **2** exhibits the lowest p*K*_a_ and delivers the largest hypochlorite over peroxide catalytic rate advantage. The fast Fe-TAML/hypochlorite catalysis is accompanied by slow noncatalytic oxidations of Orange II.

## Abstract 2

TAMLs are activated faster by H_2_O_2_ under basic conditions, and the most efficient activation is desired at near-neutral pH. The latter goal plus 100−1000-fold reactivity gain were achieved by using hypochlorite as a primary oxidant due to its lower p*K*_a_ value (7.5) compared to that of H_2_O_2_ (11.5).

## Introduction

**Chart 1:**
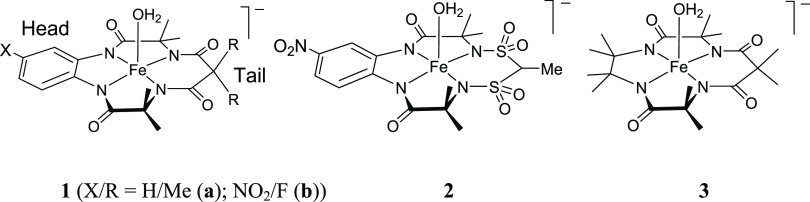
Fe-TAML Activators of Peroxides Used or Mentioned in This Work

Fe-TAML activators of peroxides are iron(III) complexes of tetraamido or bisamido bissulfonamido macrocyclic ligands.^1,2^ As presented in Chart 1, they are five-coordinate square pyramidal species in the solid state. Iron(III) is surrounded by four deprotonated amide or sulfonamide nitrogens forming a distorted plane. The fifth axial ligand is chloride, water, or alcohol. A nonwater axial ligand is replaced by a water molecule in aqueous solution to form either five-coordinate mono-aqua derivatives^3^ or six-coordinate pseudo-octahedral diaqua species after addition of the second aqua ligand.^4,5^

Fe-TAML activators **1** and **2** in Chart 1 catalyze the peroxidase-like oxidations of various substrates by primary oxidants including, *inter alia*, hydrogen and organic peroxides,^6,7^ hypochlorite,^8,9^ and under appropriate conditions, dioxygen.^10^ The simplest stoichiometric mechanism of Fe-TAML-catalyzed oxidations by H_2_O_2_ in aqueous media is straightforward.^6,11^ The iron(III) resting state of any Fe-TAML catalyst is activated by an oxidant such as H_2_O_2_ (step 1), and the active catalyst formed then attacks an electron donor, S (step 2). If S is a dye, the primary products are usually oxidized fast (step 3), which is typically not the case for more difficult-to-oxidize targets.^12,13^
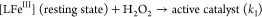
1
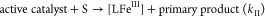
2
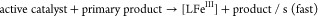
3

The mechanism given by eqs 1–3 translates into rate law 4, which resembles that in catalysis by peroxidase enzymes under the steady-state conditions (where the reverse rate constant of eq 1 is assumed to be negligible).^14,15^
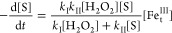
4

**Scheme 1:**
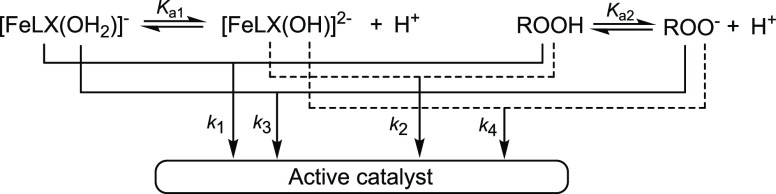
Mechanism of Reactions of Fe-TAMLs with Peroxides in Water (eq 1) that Accounts for the Bell-Shaped pH Profiles of Their Catalytic Activity X denotes either a H_2_O ligand or a coordination vacancy at iron(III).

Although Fe-TAML activators are the best known to date functional replicas of the enzymes,^2,16^ the rate constants *k*_I_ are usually significantly lower than *k*_II_ (an opposite trend holds in the enzymatic catalysis),^15^ i.e., the first step is typically rate-limiting for man-made catalysts unless the substrate is particularly difficult to oxidize. Therefore, the pH profile of the catalytic activity is predominantly determined by the acid–base properties of the catalyst and oxidants in their resting states. The first p*K*_a_’s of the aqua ligands in Fe-TAMLs cover the range of 8.4–11.4,^17^ and the p*K*_a_ of H_2_O_2_ is around 11.6.^18^ Protonated and deprotonated species react pairwise to produce the active catalyst (Scheme 1). The bell-shaped profiles of the catalytic activity appear to result primarily from the highest *k*_2_ values, where *k*_2_ is the rate constant for the interaction of the most electron-rich dianionic catalyst forms [FeLX(OH)]^2–^ with the highest reduction potential, the protonated form of the oxidant.^19^

The pH-dependent effective rate constant *k*_I_ is a function of both *K*_a1_ and *K*_a2_ (Scheme 1 and eq 5),^19^ viz., the dissociation constants of Fe-TAMLs and H_2_O_2_, respectively.
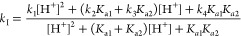
5

The value of the p*K*_a2_ of H_2_O_2_ accounts for the mild alkaline pH optimum of the catalytic activity, which is slightly shifted toward the neutral pH region by tuning the p*K*_a1_ values of Fe-TAMLs from 10.3^3^ to 8.4.^5^ The design control we have achieved thus far over the value of p*K*_a1_ has proven to be insufficient for bringing the activity maximum to circumneutral pH, a goal that remains important to maximize the technical performances of practical Fe-TAML applications such as the purification without pH adjustment of polluted waters for release to the environment.

However, the goal of achieving maximum catalytic activity at circumneutral pH can also be advanced by lowering the p*K*_a2_ value of the primary oxidant compared to hydrogen peroxide. Organic peroxides ROOH are not useful in this regard because their p*K*_a_ values are higher than that of H_2_O_2_. Not surprisingly, their use is known to shift the pH of optimal catalysis deeper into the basic region.^20^ As a first step into finding the optimal oxidant partner for maximum catalytic activity at neutral pH for our existing suite of Fe-TAMLs, we have chosen to look more deeply at hypochlorous acid (HClO), which exhibits a p*K*_a_ of 7.53,^21^ ca. four units lower than that of H_2_O_2_. Like H_2_O_2_, hypochlorous acid is also a powerful oxidant (see standard reduction potentials *E*° for half-reactions 6–9).^22^ Computer simulations assuming that eq 5 determines the overall catalytic rate for the Fe-TAML/HClO pairs show that the pH optimum of the catalytic activity should migrate to the neutral region compared with the H_2_O_2_ cases (see Figure S1 of the Supporting Information). The experimental evidence for this is described here. The extensive kinetic data presented herein for the Fe-TAML **1a-**, **1b-**, and **2**-catalyzed HClO oxidation of Orange II over a broad pH range (5–12) reveal clear rate advantages of HClO/ClO^–^ over H_2_O_2_ at circumneutral pH, which are however accompanied by minor disadvantages also discussed in this work. Thus, the goal of the present investigation was to show that using a primary oxidant with a p*K*_a_ that is significantly lower than the pK_a_ of H_2_O_2_ enables the movement of the pH of the fastest catalysis from basic pH toward neutral pH.
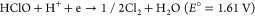
6
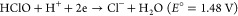
7
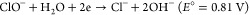
8
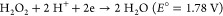
9

## Experimental Section

### Materials

Sodium acetate (99%), KH_2_PO_4_ (ACS reagent, ≥99.0%), reagent grade NaOCl solution (available chlorine, 10–15%), and Orange II (certified) were purchased from Sigma-Aldrich. Acetic acid (glacial), Na_2_HPO_4_ (anhydrous, enzyme grade), and water (HPLC grade, submicrometer-filtered) were obtained from Fisher Scientific. Na_3_PO_4_·12H_2_O was purchased from Acros Organics. Hydrogen peroxide (30%) stabilized by sodium stannate was obtained from VWR Chemicals. Fe-TAML **2** synthesized as previously described^3^ was provided by SUDOC.^23^ Complexes **1a** and **1b** were obtained from old stocks at IGS (Institute of Green Science). All the Fe-TAMLs used for kinetic measurements were purified by passing through C-18 silica gel with water as an eluent. Orange II was recrystallized from an ethanol/water mixture by adding H_2_O dropwise to its solution in warm ethanol. Other above-mentioned chemicals were used without further purification.

### Instrumentation

An Agilent 8453 UV–visible spectrophotometer with an attached temperature controller was used to perform kinetic runs. The pH of different buffers was adjusted using an Accumet Basic AB15 pH meter, which was calibrated daily using pH 4.0, 7.0, and 10.0 standard buffers.

### Sample Preparation

All buffers were prepared using HPLC grade water. Acetate buffers (10 mM; 0.01 M, pH 4.0–6.0) were prepared using NaOAc and HOAc. Phosphate buffers (0.01 M) were used at pH 6.0–8.5 and 11.0–12.0. Carbonate buffers (0.01 M) were used at pH 9.0–11.0. Stock solutions of Fe-TAMLs were made in HPLC grade water. Orange II solutions were prepared in the above-mentioned buffers. Solutions of H_2_O_2_ and NaOCl were prepared in HPLC grade water and standardized daily by UV–vis spectroscopy (ε_230 nm_ = 72.8 M^–1^ cm^–1^ for H_2_O_2_^24^ and ε_292 nm_ = 350 M^–1^ cm^–1^ for NaOCl at pH > p*K*_a_^25^).

### Kinetic Data Collection

PMMA [poly(methyl methacrylate)] cuvettes (1.0 cm) with caps (VWR) were used. Solutions of Orange II (∼50 μM) in an appropriate buffer were first added to cuvettes and kept for 5 min in the spectrometer cell holder thermostated at 25 °C. Stock solutions of Fe-TAMLs (1.5 × 10^–5^ M) were then added to achieve catalyst concentrations around 1.3 × 10^–7^ M. The reaction progress was initiated by addition of 10 μL of stock solutions of H_2_O_2_ (0.01–0.1 M) or NaOCl (6 × 10^–4^ to 0.08 M) in water. The range of concentrations of hypochlorite covered in the reaction solutions was ∼10^–5^ to 10^–4^ M. A decrease in the absorbance of Orange II was registered at 485 nm (Figure S2). Initial rates were measured in triplicate using the following pH-dependent extinction coefficients of Orange II at 485 nm: 1.9 × 10^4^ (pH 4–9.5), 1.6 × 10^4^ (pH 10.5), and 1.3 × 10^4^ M^–1^ cm^–1^ (pH 12.0). Catalytic rates (*v*_cat_) were determined by subtracting the initial rate of bleaching of Orange II by NaOCl alone from the initial rate measured in the presence of Fe-TAML. The data obtained were analyzed using SigmaPlot software (version 12.5).

### EPR Studies

The **1b** and **2** Fe^III^ complexes (0.9 mM) were buffered with 0.01 M phosphate (pH 8.2) and 15% glycerol. The spectra in the presence of H_2_O_2_ and NaClO (3 equiv for both) were obtained similarly. The signals were quantified relative to a CuEDTA spin standard. The microwave frequency was calibrated with a frequency counter and the magnetic field with an NMR gaussmeter.

## Results and Discussion

### A Key Preliminary Note

**Figure 1:**
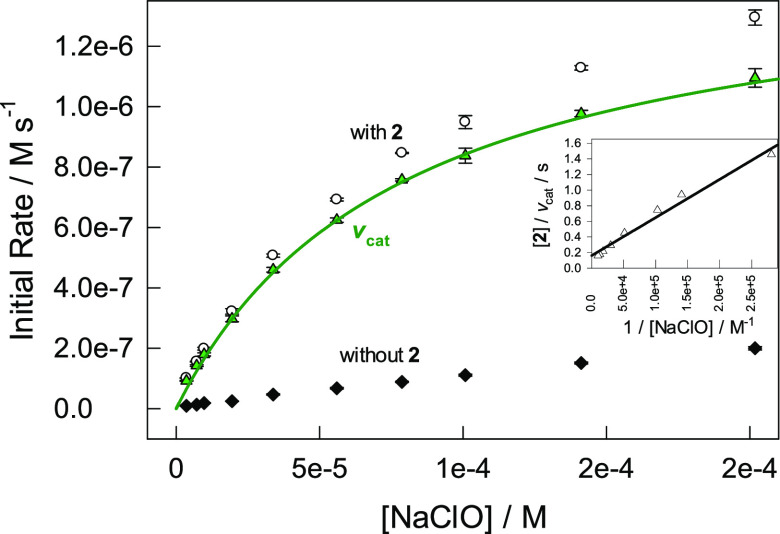
Comparison of initial rates of oxidative bleaching of Orange II (3.5 × 10^–5^ M) by NaClO with and without **2** (1.34 × 10^–7^ M) at pH 7 (0.01 M phosphate) and 25 °C. The inset shows the double inverse plot of [**2**]/*v*_cat_ vs [NaClO]^−1^ where the linear dependence supports calculation of the rate constants *k*_I_ and *k*_II_ (see eq 4 and the text for details). The solid *v*_cat_ versus [NaClO] line was calculated using the so-obtained *k*_I_ and *k*_II_ values in Table 1.

Orange II (see eq 10) is a commonly used substrate for monitoring the catalytic activity of Fe-TAML activators.^16^ It is convenient to follow its oxidation by UV–vis spectroscopy. Orange II Fe-TAML-catalyzed oxidations are subject to only minor levels of substrate inhibition, i.e., there is practically no retardation of the catalytic activity in excess concentrations of Orange II.^16,26^ Importantly, also, the noncatalyzed oxidation of Orange II by H_2_O_2_ is negligible. This is not true for the oxidation by hypochlorite,^27−30^ and therefore, the kinetic data acquired in the presence of any catalyst should be corrected for the contribution from the noncatalyzed pathway (Figure S2). The significance of the latter is highlighted in Figure 1 where the initial rates of Orange II bleaching with and without **2** (1.34 × 10^–7^ M) are compared at different NaClO concentrations.
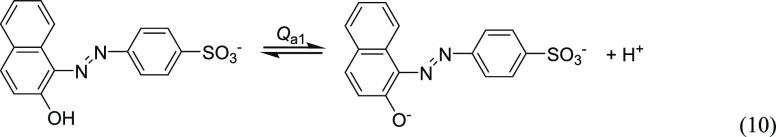
10

The measured initial rates with and without catalyst **2** differ by a factor of ca. 18 ± 2 at pH 7. The factor was obtained in the [NaClO] range of 0.3× 10^–5^ to 4.0 × 10^–5^ M where the rate is a linear function of its concentration (Figure 1). Therefore, the initial rate of the Fe-TAML-catalyzed pathway (*v*_cat_) was always calculated as the difference between the measured initial rate in the presence of a catalyst (*v*_with_) and that in its absence (*v*_without_) (eq 11).
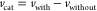
11

### Formal Kinetics

It is highly likely that the Fe-TAML-catalyzed oxidation of Orange II by NaClO follows the rate law given by eq 4. The rate of the catalytic pathway *v*_cat_ is a linear function of the **2** concentration in the range of 0.2 × 10^–7^ to 5.6 × 10^–7^ M at pH 4.0–11.5 (Figure S3). In the case of NaClO, we operate with *v*_cat_ rather than with −d[S]/d*t* as in eq 4 and it is more convenient to use the matching eq 12 instead.
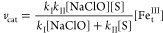
12

The data in Figure 1 show a hyperbolic dependence of *v*_cat_ on [hypochlorite], which is consistent with eq 12. The experimental data were linearized (as in Figure 1) using the Lineweaver–Burk double inverse routine ([**2**]/*v*_cat_ vs [NaClO]^−1^; see the inset in Figure 1), and the rate constant *k*_I_ was calculated from the slope of the straight line. The same process was applied to all catalysts (Table 1).

**Table 1:** Rate Constants *k*_I_ and *k*_II_ (M^–1^ s^–1^) for Orange II Bleaching by NaClO Catalyzed by **1a**, **1b**, and **2**^d^

	NaClO		H_2_O_2_
Fe-TAML	*k*_I_	*k*_II_	*k*_I_
**1a**	(1.8 ± 0.2) × 10^4^	(3.6 ± 0.1) × 10^4^	31.4 ± 0.1^a^
**1b**	(3.6 ± 0.2) × 10^5^	(3.0 ± 0.1) × 10^5^	350 ± 2^a^
**2**	(2.0 ± 0.1) × 10^5^	(5.3 ± 0.2) × 10^5^	690 ± 20^b^
			590 ± 30^c^

a From ref (31).

b From ref (3).

c This work.

d Conditions: pH 7 (0.01 M phosphate) and 25 °C. Values of *k*_I_ for H_2_O_2_ are shown for comparison.

The Table 1 data measured using NaClO and H_2_O_2_ as oxidants indicate that the values of *k*_I_ for NaClO are 2–3 orders of magnitude higher than those for H_2_O_2_.

### pH Profiles of *k*_I_ for **1a**, **1b**, and **2**

At low concentrations of NaClO, *k*_I_[NaClO] ≪ *k*_II_[S], and eq 12 becomes
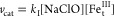
13

**Figure 2:**
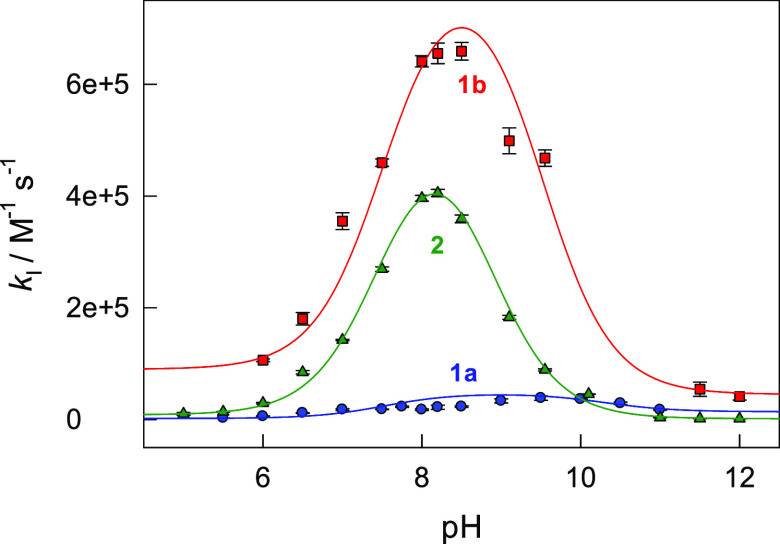
pH profiles of the rate constants *k*_I_ for **1a**, **1b**, and **2** at 25 °C, measured using different buffers (see Experimental Section). The best fits were obtained with fixed p*K*_a1_ values of 10.3, 9.5, and 8.8 for **1a**, **1b**, and **2**, respectively, and p*K*_a2_ = 7.53 for HClO. Solid lines were calculated using the best-fit values of *k*_1_–*k*_4_ summarized in Table 2.

The rates *v*_cat_ should depend linearly on [NaClO] with the slopes delivering the value for *k*_I_[Fe_t_^III^] and, hence, *k*_I_ (see Figures S4 and S5 for **1a**, **1b**, and **2** in the pH range of 5–12). The three pH profiles of *k*_I_ are presented in Figure 2. As was anticipated (Figure S1), the maximal rate constants are strongly shifted to the neutral region compared to data collected with H_2_O_2_ and are observed at pH 8.2, 8.4, and 9.0 for **2**, **1b**, and **1a**, respectively. The closest shift toward neutral pH is found for **2**, the p*K*_a1_ of which is the lowest among the three Fe-TAMLs explored herein. The highest activity in terms of *k*_I_ belongs to **1b**, the catalyst previously claimed as “an ideal Fe-TAML” from among the large suite of older tetraamido ligands.^20^ However, it is important to recognize that **2** may contain an additional decomposition mechanism in what we have termed a “kill switch” that offers considerable potential benefits in environmental applications, and the kill switch inactivates **2** faster with increasing pH.^3^ The maximum value of *k*_I_ for **1a** is seen at pH around 9.0, i.e., at a value that is considerably more basic than that reported recently by Shao et al. (ca. 7.5).^9^ Such a low value for *k*_I_ was not reached for even the much more acidic Fe-TAMLs **1b** and **2**.

The overwhelming majority of experimental data were acquired under conditions where eq 13 holds, which do not allow evaluation of the rate constants *k*_II_ as in eq 12. Therefore, we did not pursue determination/discussion of *k*_II_ values in this work. Detailed analysis of *k*_II_ is the subject of an ongoing investigation.

The data in Figure 2 agree with eq 5 derived from the mechanism in Scheme 1, in which the peroxide equilibrium ROOH ⇄ ROO^–^ + H^+^ (here R = H) is replaced by HClO ⇄ ClO^–^ + H^+^. Fitting the data to eq 5 using the p*K*_a1_ values for **1a**, **1b**, and **2** indicated in the caption of Figure 2 allows for estimation of the intrinsic rate constants *k*_1_–*k*_4_ (Table 2). Two of these (*k*_2_ and *k*_3_) are kinetically indistinguishable, and therefore, each was calculated assuming that the other counterpart equals zero. This means that *k*_2_ and *k*_3_ in Table 2 denote their upper limits.

**Table 2:** Intrinsic Rate Constants *k*_1_–*k*_4_ (M^–1^ s^–1^) for Interactions of **1a**, **1b**, and **2** and Orange II with Hypochlorite at 25 °C Calculated by Fitting the Data in Figures 2 and 4 to eq 5^a^

Fe-TAML	oxidant	10^–3^ × *k*_1_	10^–7^ × *k*_2_	10^–5^ × *k*_3_	10^–4^ × *k*_4_	reference
**1a**	NaClO	2.0 ± 0.2	2.6 ± 0.2	0.47 ± 0.03	1.4 ± 0.1	this work
**1a**	H_2_O_2_	0.13	0.0012			(19)
**1b**	NaClO	90 ± 3	7.5 ± 0.3	8.4 ± 0.2	4.5 ± 0.3	this work
**1b**	H_2_O_2_	0.4	0.0018	8	0.15	(19)
**2**	NaClO	8.5 ± 0.2	1.2 ± 0.1	6.3 ± 0.1	0.15 ± 0.03	this work
Orange II + NaClO		0.0030 ± 0.0005	0.030 ± 0.001	(4.1 ± 0.1) × 10^–4^	(4 ± 2) × 10^–5^	this work

a The complementary data for H_2_O_2_ are included for comparison.

As noted above, the values of *k*_I_ are 2–3 orders of magnitude higher for hypochlorite than for H_2_O_2_. A similar trend holds for the individual rate constants *k*_1_–*k*_4_ (Table 2). The upper limits for *k*_2_ of 1.2 × 10^7^ to 7.5 × 10^7^ M^–1^ s^–1^ are consistent with the anticipated fastest interaction between HClO as the most powerful oxidizing species (cf. half-reactions 6 and 8) and the deprotonated dianionic Fe-TAML species [FeLX(OH)]^2–^ (Scheme 1) as the easiest-to-oxidize, most electron-rich catalyst species. The other three individual rate constants (*k*_1_, *k*_3_ and *k*_4_) are expectedly lower.

### Hydrogen Peroxide and Hypochlorite Generate Similar Species in mM Solutions According to EPR

**Figure 3:**
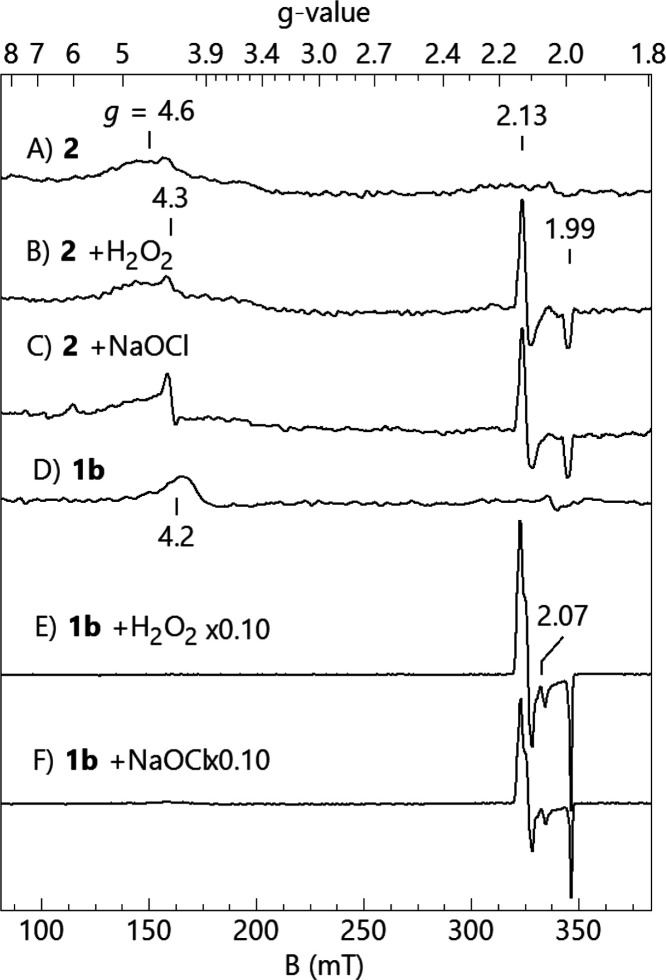
EPR spectra of **1b** and **2** (0.9 × 10^–3^ M) before and after adding H_2_O_2_ and hypochlorite (2.7 × 10^–3^ M) at pH 8.2 (0.01 M phosphate) in the presence of 15% glycerol. The signal at *g* = 4.3 is a minor ferric impurity. The signal at *g* = 2.07 is an unknown minor species not previously observed after oxidation. The scales for E and F are reduced by 10 compared to those for B and C. Sample temperature, 17 K; microwaves, 0.2 mW at 9.631 GHz.

The similarities of the kinetics of Fe-TAML-catalyzed oxidation by H_2_O_2_ and NaClO prompted us to look at species produced in aqueous solution in the presence of these oxidants. The species generated from **1b** and **2** at pH 8.2, at which the highest *k*_I_ for **2** was recorded, were investigated by EPR spectroscopy (Figure 3). As previously noted, **1b** and **2** show broad signals at *g* = 4.6 and 4.2, respectively, that sharpen in the presence of glycerol. These signals are attributed to intermediate spin, *S* = 3/2 ferric-TAMLs, where the broadness of the signals is thought to result from intermolecular interaction associated with Fe-TAML stacking in solution. In some previously studied Fe-TAML complexes, glycerol significantly reduces the amount of this stacking and spin quantification gives species concentrations that agree with the added concentration of iron in solution. However, for **2**, spin quantification of the observed *S* = 3/2 signal accounts for only 10% of the iron, with the majority of the signal too broad to accurately quantify.

The addition of 3 equiv of either oxidant, H_2_O_2_ or NaClO, caused a new species to be observed for both **1b** and **2**. The electronic properties of the *S* = 1/2 species are independent of the oxidant. The new species has *S* = 1/2 with *g* = (2.13, 2.11, 1.99) and has been observed previously from formation of the mixed-valent dimer Fe^III^–O–Fe^IV^ complex of Fe-TAML.^10^ Spin quantification indicated a minor amount of reaction for **2** with an oxidant, with nearly no loss of the *S* = 3/2 precursor species and 3% of the *S* = 1/2 species. However, for **1b**, the *S* = 1/2 species accounted for 60% of the iron in the sample.

The EPR data were obtained at mM concentrations of Fe-TAMLs at 13 K in the presence of 15% glycerol, which are very different reaction conditions than the nM glycerol-free solutions used for catalytic applications in pure water. The variation in concentrations of 5–6 orders of magnitude may affect both dimerization/oligomerization and electron/atom transfer processes between participating species of the various oxidation states.^2^ Thus, although the EPR spectra look similar for NaClO and H_2_O_2_, this does not rule out that different species may exist under dilute conditions.

### Comparison of the Intrinsic Rate Constants for the Uncatalyzed and Fe-TAML-Catalyzed Oxidation of Orange II by Hypochlorite

**Scheme 2:**
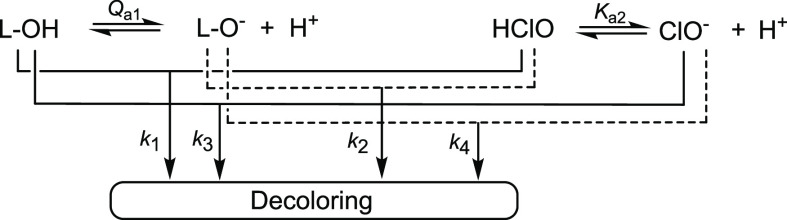
Mechanism of Oxidation of Orange II (L-OH) by Hypochlorite in Water Consistent with the Bell-Shaped pH Profile of the Observed Second-Order Rate Constant *k*_II_′ in Figure 4 L-O^–^ denotes the deprotonated form of the dye (eq 10).

The catalyzed values of *k*_1_–*k*_4_ in Table 2 (Scheme 1) are worth comparing with the related uncatalyzed values for the direct oxidation of Orange II by hypochlorite (bottom of Table 2). The results of kinetic studies of the uncatalyzed process including its pH dependence have been reported,^28,30^ although the intrinsic rate constants *k*_1_–*k*_4_ were not calculated. The mechanism of Orange II oxidation by hypochlorite presented in Scheme 2 is formally similar to that in Scheme 1, since both Fe-TAML (Scheme 1) and the dye (eq 10) undergo a single deprotonation and both forms react with HClO and ClO^–^. As demonstrated below, the intrinsic rate constants *k*_1_–*k*_4_ for the two processes serve as parameters for intimately comparing the efficacy of the catalyzed and noncatalyzed oxidation of Orange II.

**Figure 4:**
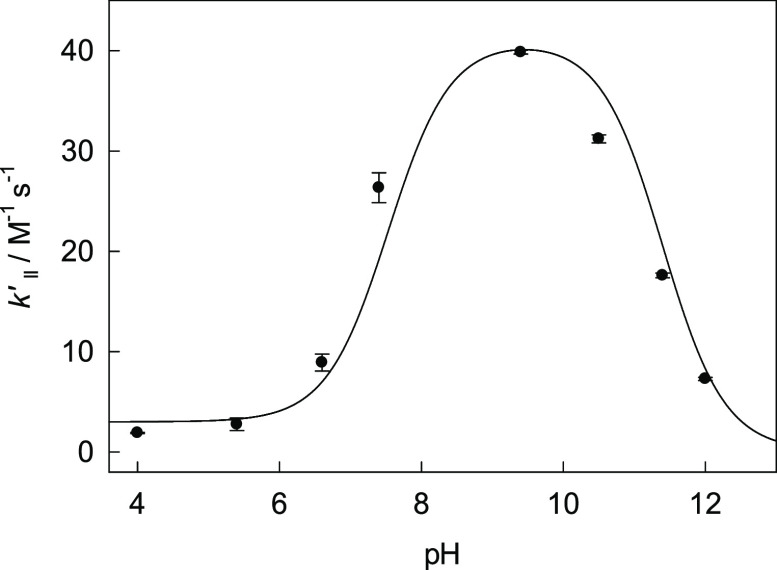
pH profile for the rate constant *k*_II_′ at 25 °C. Measurements at pH 4.0 and 5.6; 6.6, 7.4, and 12.0; and 9.4, 10.5, and 11.4 were made in 0.01 M acetate, phosphate, and carbonate buffers, respectively. The solid line was calculated using the best-fit values of *k*_1_–*k*_4_ summarized in Table 2.

We have found that the pseudo-first-order rate constants *k*_obs_ for the bleaching of Orange II by hypochlorite measured in excess hypochlorite depend linearly on the oxidant concentration at all pH values measured (4–12, Figure S6). The slopes, i.e., the second-order rate constants *k*_II_′, have a bell-shaped pH dependence (Figure 4), which is consistent with eq 14 derived from Scheme 2:
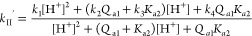
14

Equation 14 is identical to eq 5, in which *K*_a1_ is replaced by *Q*_a1_. Fitting the data to eq 14 using the p*Q*_a1_ and p*K*_a2_ values of 11.4^30^ and 7.53,^21^ respectively, provided a set of rate constants *k*_1_–*k*_4_, with *k*_2_ and *k*_3_, as above, being kinetically indistinguishable. All rate constants are summarized in Table 2.

The pH profile in Figure 4 is broader than that in Figure 2 due to the larger p*Q*_a1_ value for Orange II (11.4) than those of Fe-TAMLs **1b** (9.3) and **2** (8.8). The rate constants *k*_1_–*k*_4_ are noticeably higher in the case of Fe-TAMLs compared with Orange II, accounting for faster Fe-TAML-catalyzed oxidation. The value of *k*_2_ is the largest—the HClO/L-O^–^ pair is the most reactive (cf. with the related argumentation for the HClO/[FeLX(OH)]^2–^ pair presented above). The broad maximum in Figure 4 allows one to easily estimate p*K*_a_ values of both participants, i.e., HClO and Orange II, using the tangent routine (see Figure S7) as often used in enzymology.^32^ The values of p*Q*_a1_ and p*K*_a2_ of 11.2 and 7.4 compare well with those indicated above.

### Comments on Degradation of Fe-TAMLs by Hypochlorite

**Figure 5:**
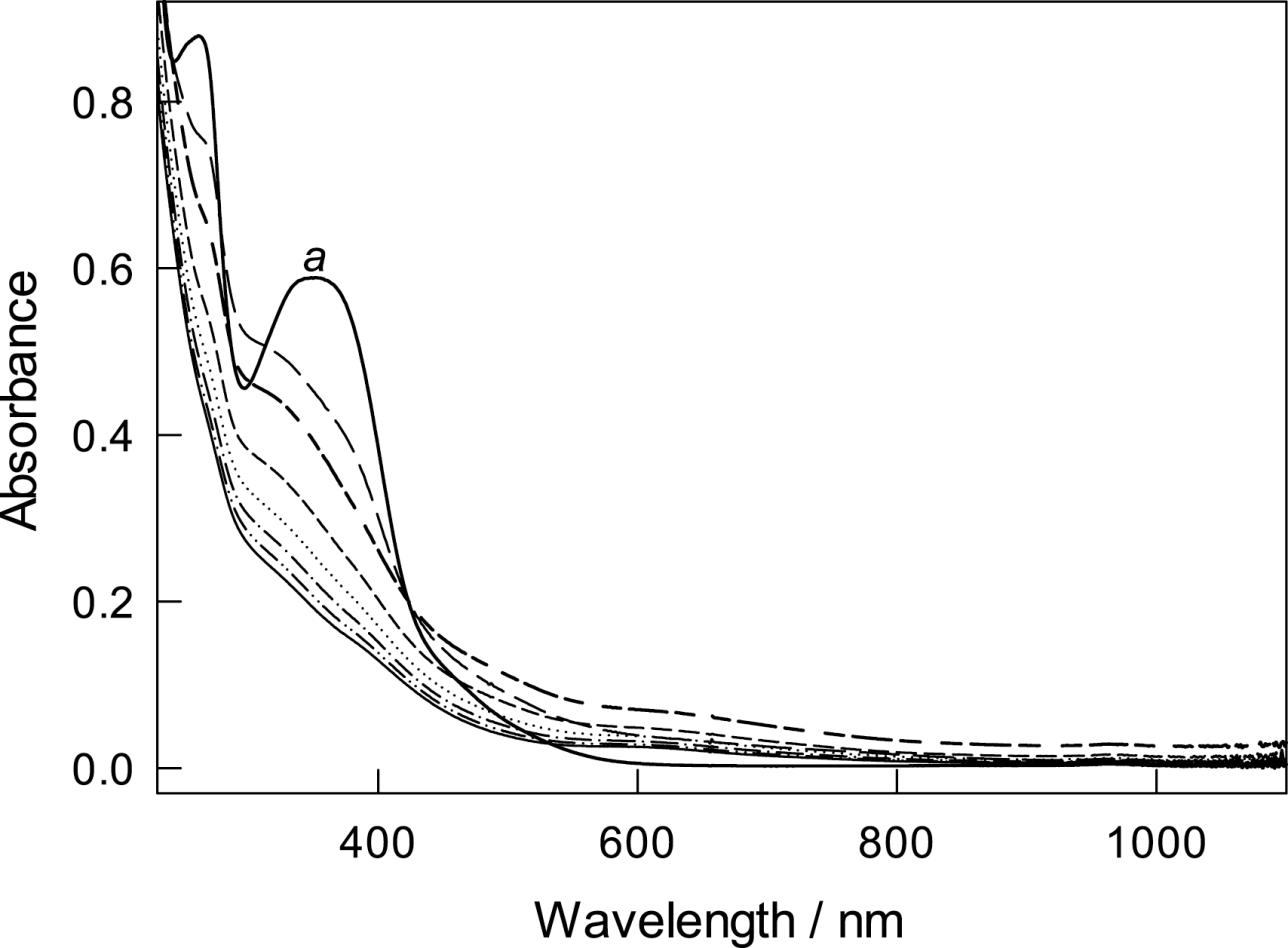
Spectral changes of **2** (5.8 × 10^–5^ M) in the presence of 45 × 10^–5^ M NaClO at pH 7 (0.01 M phosphate) and 25 °C. (*a*) Spectrum of **2** prior to adding NaClO; other spectra were obtained every 5 s.

The present story would not be complete without mentioning that **2** is degraded rather rapidly by NaClO at pH < 7 (Figure 5), with activators **1a** and **1b** being more resistant to hypochlorite (Figures S8 and S9). This may be limited using **2** with NaClO under acidic conditions because the catalytic oxidation vanishes rapidly due to catalyst inactivation and the process is then dominated by the slower noncatalytic process involving hypochlorite only. This inactivation affects none of our conclusions on the catalytic activity of **2** and calculation of the rate constants *k*_I_. The guarantees are the accurate calculation of *v*_cat_ using eq 11 combined with initial rate measurements where at early stages of Orange II bleaching, catalyst degradation is just starting. The degradation precludes evaluating the operational stability of **1a**, **1b**, and **2** in hypochlorite processes in terms of *k*_i_ (the rate constant for intramolecular inactivation of the active catalyst, active catalyst → inactive form, *k*_i_) using the routine developed for H_2_O_2_ as an oxidant.^33,34^ The routine requires that the substrate is degraded only by the catalytic process and resistant to the chosen oxidant in the absence of the catalyst. Such a test substrate is difficult to find when NaClO is the oxidant.

More careful inspection of the spectra in Figure 5 indicates that a decrease in absorbance at 355 nm (maximum of **2**) occurs with increasing optical density at 600–1000 nm, which is indicative of the formation of iron(IV) species. This observation is consistent with the EPR data presented above. Both 355 nm and 600–1000 nm bands fade eventually with time. A mechanistically challenging problem here is how **2** gets oxidized. The mechanism of oxidation of Fe-TAMLs by hypochlorite is a subject of ongoing investigation.

## Conclusions

The results of this study provide a convincing verification of the first step of Fe-TAML catalyzed oxidations, which we have thought was operating for over three decades and where detailed kinetic studies focused on the mechanism of Scheme 1 were first prominently published in 2008.^19^ The study of the mechanism of Fe-TAML catalytic processes centered on Scheme 1 that relies on the steady-state approximation has added to the understanding derived from iterative catalyst design^35^ that has been a continuous theme in the Fe-TAML program for four decades. The application of mechanistic studies focused on Scheme 1 and the many newly developed tangents have made clear how to use the acid–base properties of appropriate oxidizing agents to also shift the pH of maximum catalytic activity under *k*_I_-controlled conditions toward neutral pH. This combination of the intuitively driven iterative design protocol that has created all the Fe-TAML catalysts for subsequent mechanistic studies with mechanistic studies based on the steady-state approximation that perfectly quantify the powers of the design insight has amounted to a powerful program in seeking catalysts with useful technical performances for environmental applications. This study also shows that Fe-TAML/hypochlorite displays catalysis that is 2–3 orders of magnitude faster than Fe-TAML/peroxide under *k*_I_-controlled conditions.

## Supporting Information Available

Calculated theoretical reactivity–pH profiles for Fe-TAMLs with different oxidants, spectral changes of Orange II under the catalytic conditions, dependencies of *v*_cat_ vs [**2**] and [NaClO], *k*_obs_ vs [NaClO] for direct oxidation of Orange II by hypochlorite, tangent routine for calculating p*K*_a_ values, and spectral changes of **1a** and **1b** induced by NaClO (PDF)

## References

[ref1] Collins T. J. TAML oxidant activators: a new approach to the activation of hydrogen peroxide for environmentally significant problems. Acc. Chem. Res. 2002, 35, 782–790. 10.1021/ar010079s. 12234208

[ref2] Collins T. J.; Ryabov A. D. Targeting of high-valent iron-TAML activators at hydrocarbons and beyond. Chem. Rev. 2017, 117, 9140–9162. 10.1021/acs.chemrev.7b00034. 28488444

[ref3] Warner G. R.; Somasundar Y.; Jansen K. C.; Kaaret E. Z.; Weng C.; Burton A. E.; Mills M. R.; Shen L. Q.; Ryabov A. D.; Pros G.; Pintauer T.; Biswas S.; Hendrich M. P.; Taylor J. A.; Vom Saal F. S.; Collins T. J. Bioinspired, multidisciplinary, iterative catalyst design creates the highest performance peroxidase mimics and the field of sustainable ultradilute oxidation catalysis. ACS Catal. 2019, 9, 7023–7037. 10.1021/acscatal.9b01409.

[ref4] Ghosh A.; Ryabov A. D.; Mayer S. M.; Horner D. C.; Prasuhn D. E.; Sen Gupta S.; Vuocolo L.; Culver C.; Hendrich M. P.; Rickard C. E. F.; Norman R. E.; Horwitz C. P.; Collins T. J. Understanding the mechanism of H+-induced demetalation as a design strategy for robust iron(III) peroxide-activating catalysts. J. Am. Chem. Soc. 2003, 125, 12378–12378. 10.1021/ja0367344. 14531659

[ref5] Ellis W. C.; Tran C. T.; Roy R.; Rusten M.; Fischer A.; Ryabov A. D.; Blumberg B.; Collins T. J. Designing green oxidation catalysts for purifying environmental waters. J. Am. Chem. Soc. 2010, 132, 9774–9781. 10.1021/ja102524v. 20565079 PMC2925145

[ref6] Ryabov A. D.; Collins T. J. Mechanistic considerations on the reactivity of green FeIII-TAML activators of peroxides. Adv. Inorg. Chem. 2009, 61, 471–521. 10.1016/S0898-8838(09)00208-6.

[ref7] Ryabov A. D. Green challenges of catalysis via Iron(IV)oxo and Iron(V)oxo species. Adv. Inorg. Chem. 2013, 65, 118–163. 10.1016/B978-0-12-404582-8.00004-3.

[ref8] Tang L. L.; DeNardo M. A.; Schuler C. J.; Mills M. R.; Gayathri C.; Gil R. R.; Kanda R.; Collins T. J. Homogeneous catalysis under ultradilute conditions: TAML/NaClO oxidation of persistent metaldehyde. J. Am. Chem. Soc. 2017, 139, 879–887. 10.1021/jacs.6b11145. 28045254

[ref9] Shao B.; Zhu J.; Zhou G.; Pan B.; Zhang X.; Dong L.; Guan X. Importance of high-valent iron complex and reactive radicals in organic contaminants’ abatement by the Fe-TAML/free chlorine system. ACS ES&T Eng. 2021, 1, 1401–1409. 10.1021/acsestengg.1c00124.

[ref10] Tang L. L.; Gunderson W. A.; Weitz A. C.; Hendrich M. P.; Ryabov A. D.; Collins T. J. Activation of dioxygen by a TAML activator in reverse micelles: Characterization of an Fe^III^Fe^IV^ dimer and associated catalytic chemistry. J. Am. Chem. Soc. 2015, 137, 9704–9715. 10.1021/jacs.5b05229. 26161504 PMC5286568

[ref11] Chahbane N.; Popescu D.-L.; Mitchell D. A.; Chanda A.; Lenoir D.; Ryabov A. D.; Schramm K.-W.; Collins T. J. Fe^III^-TAML-catalyzed green oxidative degradation of the azo dye Orange II by H2O2 and organic peroxides: products, toxicity, kinetics, and mechanisms. Green Chem. 2007, 9, 49–57. 10.1039/B604990G.

[ref12] Somasundar Y.; Burton A. E.; Mills M. R.; Zhang D. Z.; Ryabov A. D.; Collins T. J. Quantifying evolving toxicity in the TAML/peroxide mineralization of propranolol. iScience 2021, 24, 101897 10.1016/j.isci.2020.101897. 33364585 PMC7753967

[ref13] Frame H. C.; Somasundar Y.; Warner G. R.; Ryabov A. D.; Collins T. J. Kinetics of catalytic oxidation of the potent aquatic toxin microcystin-LR by latest generation TAML activators. J. Coord. Chem. 2020, 73, 2613–2620. 10.1080/00958972.2020.1840562.

[ref14] Dunford H. B. Peroxidases. Adv. Inorg. Biochem. 1982, 4, 41–80. 10.1016/S0009-2797(00)00201-5.

[ref15] Dunford H. B. Heme Peroxidases; Wiley-VCH: NY, Chichester, Weinheim, 1999.

[ref16] Ryabov A. D. Mechanistic Puzzles from iron(III) TAML activators including substrate inhibition, zero-order and dual catalysis. Adv. Inorg. Chem. 2021, 78, 183–225. 10.1016/bs.adioch.2020.12.005.

[ref17] Mills M. R.; Weitz A. C.; Zhang D. Z.; Hendrich M. P.; Ryabov A. D.; Collins T. J. A “beheaded” TAML Activator: a compromised catalyst that emphasizes the linearity between catalytic activity and p*K*_a_. Inorg. Chem. 2016, 55, 12263–12269. 10.1021/acs.inorgchem.6b01988. 27934426 PMC5479581

[ref18] Jones C. W. Applications of hydrogen peroxide and derivatives; The Royal Society of Chemistry: Cambridge, 1999, p. 39, 10.1039/9781847550132.

[ref19] Ghosh A.; Mitchell D. A.; Chanda A.; Ryabov A. D.; Popescu D. L.; Upham E.; Collins G. J.; Collins T. J. Catalase-peroxidase activity of iron(III)-TAML activators of hydrogen peroxide. J. Am. Chem. Soc. 2008, 130, 15116–15126. 10.1021/ja8043689. 18928252

[ref20] Popescu D.-L.; Chanda A.; Stadler M. J.; Mondal S.; Tehranchi J.; Ryabov A. D.; Collins T. J. Mechanistically inspired design of Fe^III^-TAML peroxide-activating catalysts. J. Am. Chem. Soc. 2008, 130, 12260–12261. 10.1021/ja805099e. 18722448

[ref21] Smith R. M.; Martell A. E. Critical Stability Constants; 4*,* Plenum Press: NY, 1976.

[ref22] Lide D. R., CRC Handbook of Chemistry and Physics; 87th ed., CRC Taylor & Francis: New York, 2006–2007.

[ref23] Website: https://www.sudoc.com.

[ref24] George P. The chemical nature of the second hydrogen peroxide compound formed by cytochrome c peroxidase and horseradish peroxidase. Biochem. J. 1953, 54, 267–276. 10.1042/bj0540267. 13058869 PMC1268935

[ref25] Kelm M.; Pashalidis I.; Kim J. I. Spectroscopic investigation on the formation of hypochlorite by alpha radiolysis in concentrated NaCl solutions. Appl. Radiat. Isot. 1999, 51, 637–642. 10.1016/S0969-8043(99)00113-X.

[ref26] Somasundar Y.; Shen L. Q.; Hoane A. G.; Tang L. L.; Mills M. R.; Burton A. E.; Ryabov A. D.; Collins T. J. Structural, mechanistic and ultra-dilute catalysis portrayal of substrate inhibition in the TAML–hydrogen peroxide catalytic oxidation of the persistent drug and micropollutant, propranolol. J. Am. Chem. Soc. 2018, 140, 12280–12289. 10.1021/jacs.8b08108. 30180543

[ref27] Oakes J.; Gratton P. Kinetic investigations of the oxidation of arylazonaphthol dyes in hypochlorite solutions as a function of pH. J. Chem. Soc., Perkin Trans. 1998, 10, 2201–2206. 10.1039/a805949g.

[ref28] Oakes J.; Gratton P. Kinetic investigations of azo dye oxidation in aqueous media. J. Chem. Soc., Perkin Trans. 1998, 9, 1857–1864. 10.1039/a803892i.

[ref29] Oakes J.; Gratton P.; Gordon-Smith T. Combined kinetic and spectroscopic study of oxidation of azo dyes in surfactant solutions by hypochlorite. Dyes Pigm. 2000, 46, 169–180. 10.1016/S0143-7208(00)00051-6.

[ref30] Urano H.; Fukuzaki S. The mode of action of sodium hypochlorite in the decolorization of azo dye Orange II in aqueous solution. Biocontrol Sci. 2011, 16, 123–126. 10.4265/bio.16.123. 21946323

[ref31] DeNardo M. A.; Mills M. R.; Ryabov A. D.; Collins T. J. Unifying evaluation of the technical performances of iron-tetra-amido macrocyclic ligand oxidation catalysts. J. Am. Chem. Soc. 2016, 138, 2933–2936. 10.1021/jacs.5b13087. 26886296

[ref32] Ryabov A. D. Practical Kinetics and Mechanisms of Chemical and Enzymatic Reactions; *2021. ISBN: 1–5275–6212-3; ISBN13: 978–1–5275-6212-7.* Cambridge Scholars Publishing, Lady Stephenson Library: Newcastle upon Tyne, NE6 2PA, UK, 2021.

[ref33] Chanda A.; Ryabov A. D.; Mondal S.; Alexandrova L.; Ghosh A.; Hangun-Balkir Y.; Horwitz C. P.; Collins T. J. The activity-stability parameterization of homogeneous green oxidation catalysts. Chem. – Eur. J. 2006, 12, 9336–9345. 10.1002/chem.200600630. 17029311

[ref34] Emelianenko M.; Torrejon D.; Denardo M. A.; Ryabov A. D.; Collins T. J. Estimation of rate constants in nonlinear reactions involving chemical inactivation of oxidation catalysts. J. Math. Chem. 2014, 52, 1460–1476. 10.1007/s10910-014-0322-4.

[ref35] Collins T. J. Designing ligands for oxidizing complexes. Acc. Chem. Res. 1994, 27, 279–285. 10.1021/ar00045a004.

