# Supplementary material for: The Mechanism of Formation of Active Fe-TAMLs Using HClO Enlightens Design for Maximizing Catalytic Activity at Environmentally Optimal, Circumneutral pH
Source: Inorg Chem. 2023 Mar 27;62(14):5586–92. doi: 10.1021/acs.inorgchem.3c00104 (PMC10091481; doi:10.1021/acs.inorgchem.3c00104)
Supplement: Supplementary file 1 — ic3c00104_si_001.pdf [file ic3c00104_si_001.pdf]

Supporting Information

The mechanism of formation of active Fe-TAMLs using HClO enlightens design for maximizing catalytic activity at environmentally optimal, circumneutral pH

Parameswar Pal, Marcus C. Schafer, Michael P. Hendrich, Alexander D. Ryabov, Terrence J. Collins**

Institute for Green Science, Department of Chemistry, Carnegie Mellon University, 4400 Fifth Avenue, Pittsburgh, PA 15213, USA

AUTHOR INFORMATION

Corresponding Authors

E-mail: ryabov@andrew.cmu.edu. E-mail: tc1u@andrew.cmu.edu.

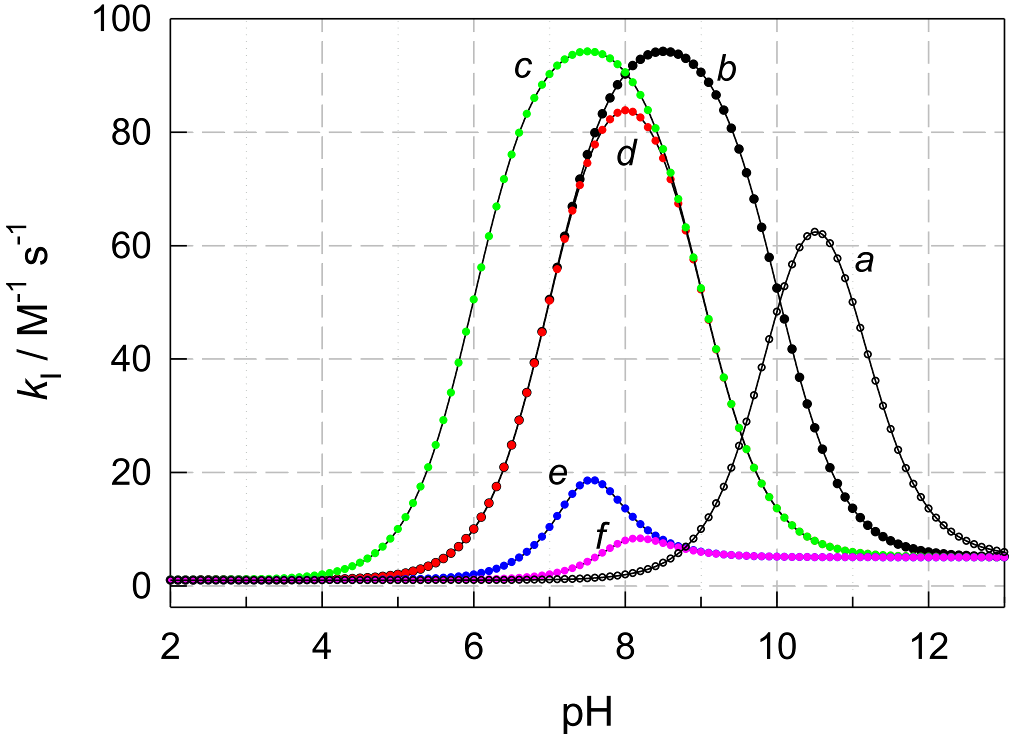

Figure S1. Calculated pH profiles for the rate constants k_I using eq 5 using fixed arbitrary values of the rate constants (k_1 = 5, k_3 = 0 and k_4 = 5 M^{-1} s^{-1}) and variable values of (k_2, M^{-1} s^{-1}; pK_{a1} and pK_{a2}) the meaning of which is shown in Scheme 1: ***a*** (1000; 10; 11), ***b*** (100; 7; 10), ***c*** (100; 6; 9), ***d*** (100; 7; 9), ***e*** (1000; 6; 9) and ***f*** (1000; 6; 10).

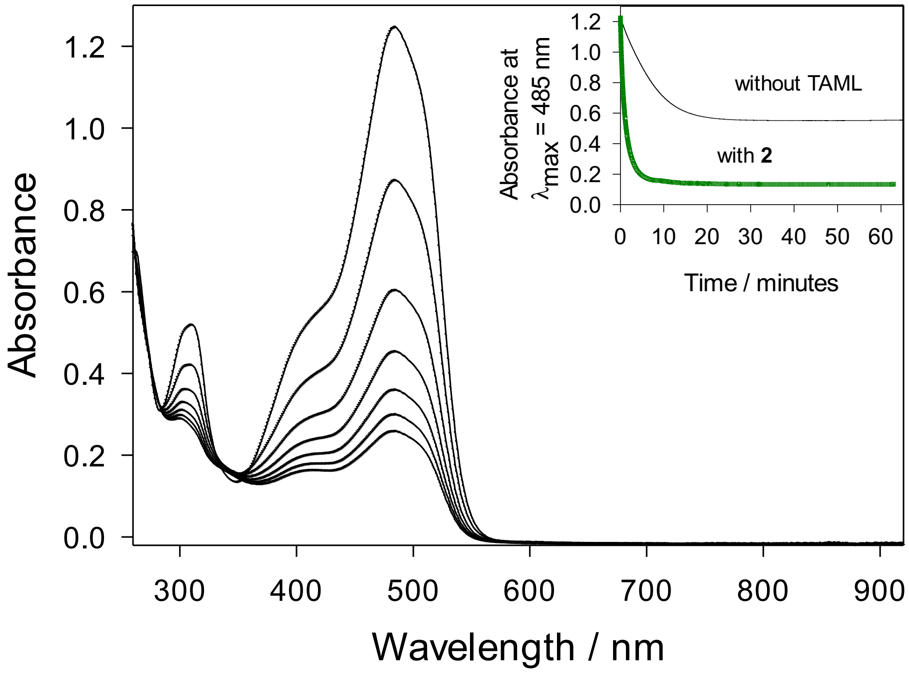

Figure S2. Spectral changes of Orange II (7×10^{-5} M) induced by NaOCl (1.1×10^{-4} M) in the presence of **2** (1.5×10^{-7} M) at pH 7 (0.01 M phosphate) and 25 ºC. Spectra were recorded each 30 s. Inset compares the speed of oxidation with and without **2**.

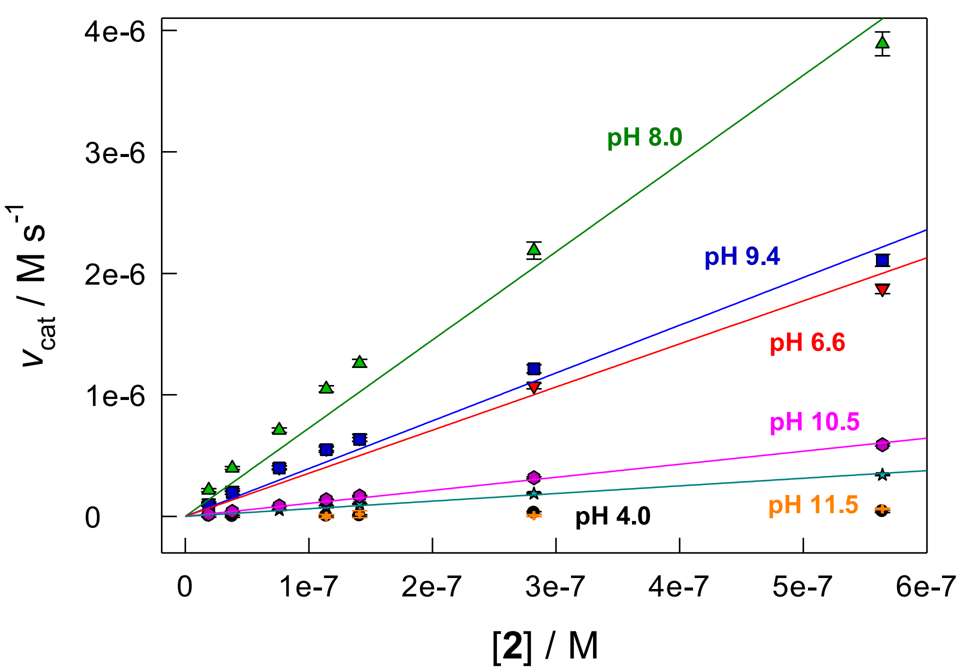

Figure S3. Dependence of v_{cat} on different concentrations of **2** in the pH range from 4.0 to 11.5. Conditions: [NaClO] = 1.1 $\times10^{-4}$ M, [Orange II] = 5.5 $\times10^{-5}$ M, 298 K. Reasons for minor deviations from the linearity observed at pH 8.0 are discussed in detail in reference S1. They are likely associated with either ground state iron(III)-TAML aggregation suggested by the EPR data or dimerization of the iron(IV)oxo intermediate generated from **2** and hypochlorite.

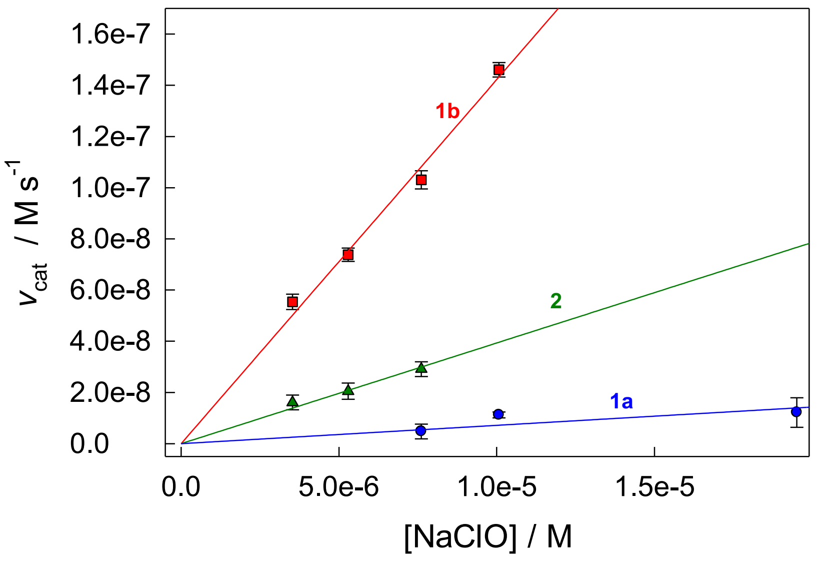

Figure S4. v_{cat} as a function of [NaClO] at low concentrations of the oxidant when $v_{cat} = k_I[\text{NaClO}][\text{Fe}_t^{III}]$. Conditions: [**1a**] = [**1b**] = [**2**] = 1.3 ×10^{-7} M, [Orange II] = 3.5 ×10^{-5} M, pH 6.0 (0.01 M acetate), 298 K.

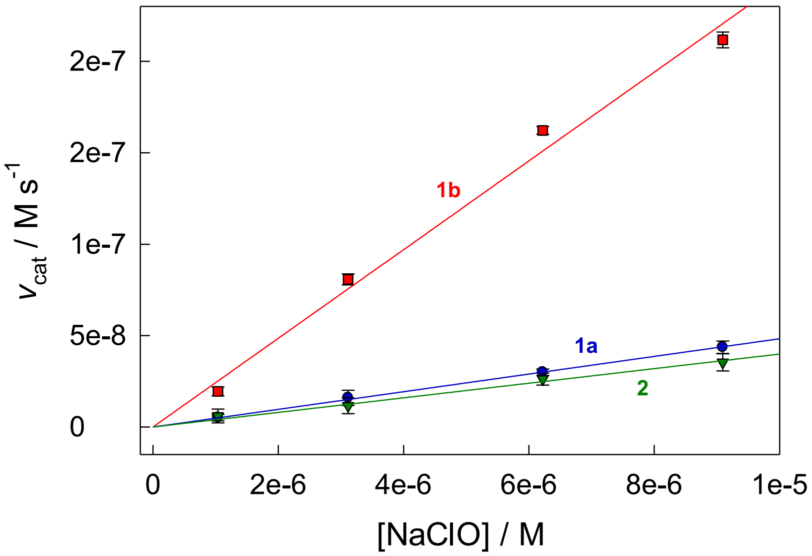

Figure S5. v_{cat} as a function of [NaClO] at low concentrations of the oxidant when $v_{\mathrm{cat}} = k_{\mathrm{I}}[\mathrm{NaClO}][\mathrm{Fe}_{\mathrm{t}}^{\mathrm{III}}]$. Conditions: [**1a**] = [**1b**] = [**2**] = 1.3 ×10^{-7} M, [Orange II] = 3.5 ×10^{-5} M, pH 10.0 (0.01 M Carbonate), 298 K.

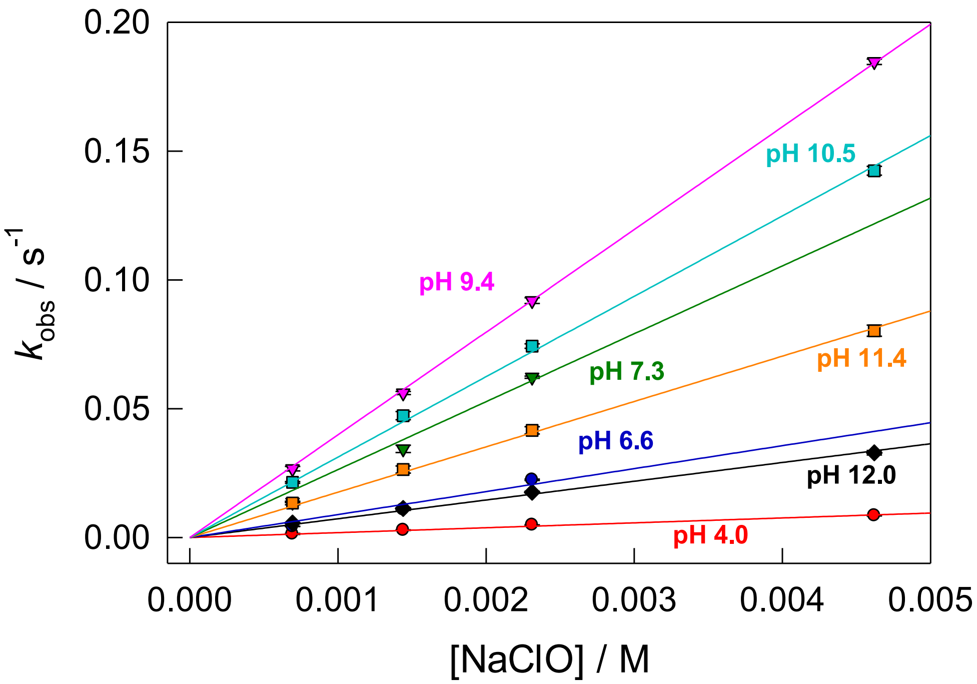

Figure S6. Pseudo-first-order rate constants k_{obs} for non-catalytic oxidation of Orange II by hypochlorite against [NaClO] at different pH. Conditions: [Orange II] = 3.5 ×10^{-5} M, [NaClO] 6.9 ×10^{-4} – 4.6 ×10^{-3} M, 298 K. Values of k_{obs} were obtained as the slope of the lines ln[Orange II]$_{t=t}$ = ln[Orange II]$_{t=0}$ - k_{obs} × t.

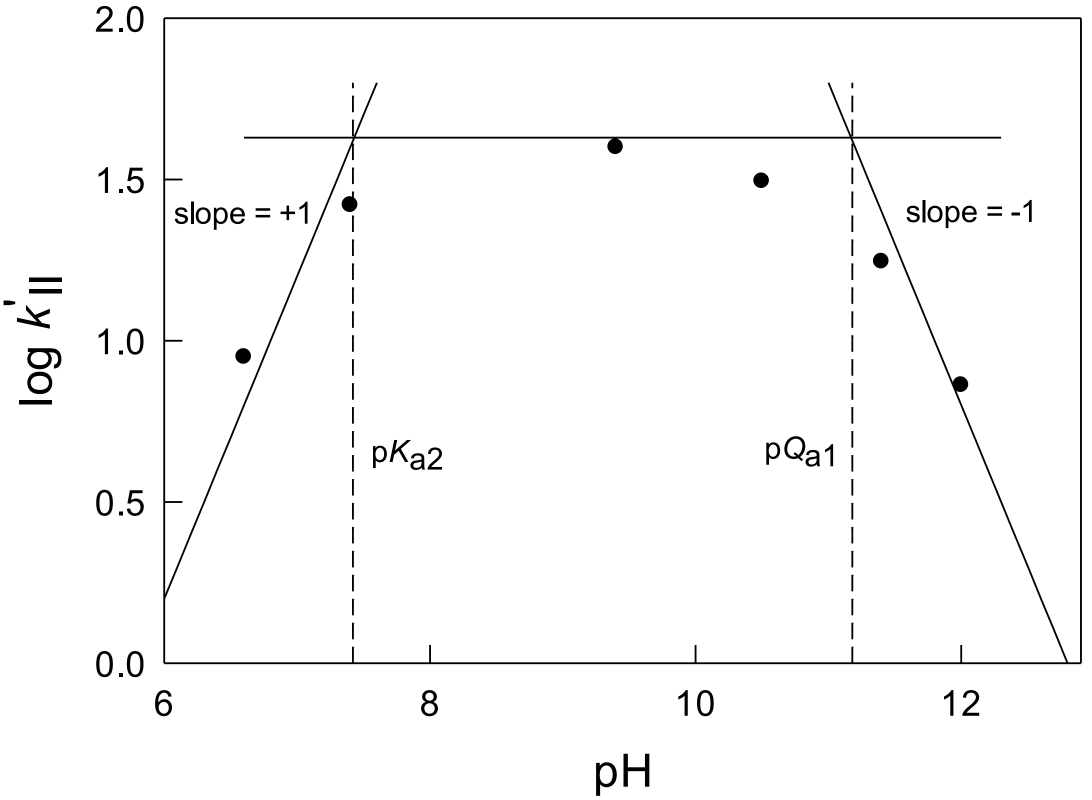

Figure S7. The tangent routine for obtaining pK_a values of HOCl and Orange II from the kinetic data presented in Figure 4 as described in reference 32. The left part of the plot has a slope of +1, since $k'_{II} = k_3K_{a2}/[H^+]$ and log k'_{II} = constant + pH in this pH range. The right part is a straight line with the slope = −1, since log k'_{II} = constant – pH.

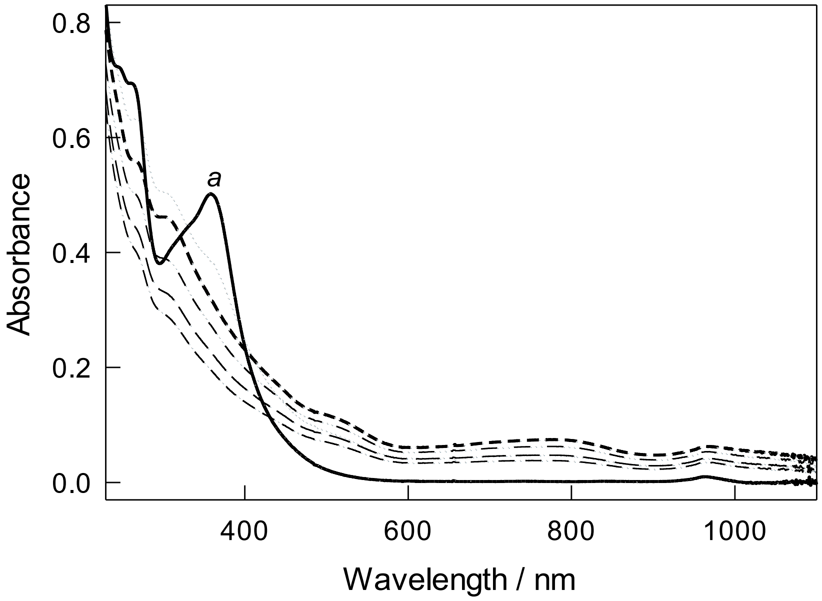

Figure S8. Spectral changes of **1b** (5.5×10^{-5} M) in the presence of 4.2×10^{-4} M of NaClO at pH 7 (0.01 M phosphate) and 25 °C. (**a**) Spectrum of **1b** prior to adding NaClO; other spectra were obtained after 5, 10, 20, 35, and 50 sec after addition.

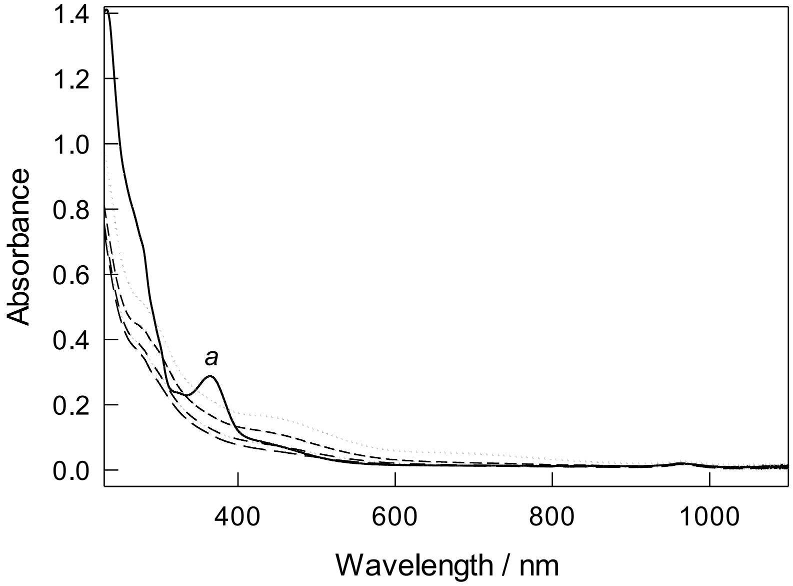

Figure S9. Spectral changes of **1a** (5.3 $\times 10^{-5}$ M) in the presence of 4.1 $\times 10^{-4}$ M of NaClO at pH 7 (0.01 M phosphate) and 25 °C. (**a**) Spectrum of **1a** prior to adding NaClO; other spectra were obtained after 10, 20, 40, and 60 s after addition.

References in Supporting Information

1S. A. D. Ryabov "Mechanistic Puzzles from Iron(III) TAML Activators Including Substrate Inhibition, Zero-Order and Dual Catalysis". *Adv. Inorg. Chem.*, 2021, *78*, 183-225.

2S. A. D. Ryabov "Practical Kinetics and Mechanisms of Chemical and Enzymatic Reactions" Cambridge Scholars Publishing, Lady Stephenson Library, Newcastle upon Tyne, NE6 2PA, UK, 2021. ISBN: 1-5275-6212-3; ISBN13: 978-1-5275-6212-7.
